# HGF Mediates the Anti-inflammatory Effects of PRP on Injured Tendons

**DOI:** 10.1371/journal.pone.0067303

**Published:** 2013-06-28

**Authors:** Jianying Zhang, Kellie K. Middleton, Freddie H. Fu, Hee-Jeong Im, James H-C. Wang

**Affiliations:** 1 MechanoBiology Laboratory, Departments of Orthopaedic Surgery, Bioengineering, and Mechanical Engineering and Materials Science, University of Pittsburgh, Pittsburgh, Pennsylvania, United States of America; 2 Departments of Biochemistry and Internal Medicine Rush University Medical Center, Chicago, Illinois, United States of America; Universidade de São Paulo, Brazil

## Abstract

Platelet-rich plasma (PRP) containing hepatocyte growth factor (HGF) and other growth factors are widely used in orthopaedic/sports medicine to repair injured tendons. While PRP treatment is reported to decrease pain in patients with tendon injury, the mechanism of this effect is not clear. Tendon pain is often associated with tendon inflammation, and HGF is known to protect tissues from inflammatory damages. Therefore, we hypothesized that HGF in PRP causes the anti-inflammatory effects. To test this hypothesis, we performed *in vitro* experiments on rabbit tendon cells and *in vivo* experiments on a mouse Achilles tendon injury model. We found that addition of PRP or HGF decreased gene expression of COX-1, COX-2, and mPGES-1, induced by the treatment of tendon cells *in vitro* with IL-1β. Further, the treatment of tendon cell cultures with HGF antibodies reduced the suppressive effects of PRP or HGF on IL-1β-induced COX-1, COX-2, and mPGES-1 gene expressions. Treatment with PRP or HGF almost completely blocked the cellular production of PGE_2_ and the expression of COX proteins. Finally, injection of PRP or HGF into wounded mouse Achilles tendons *in vivo* decreased PGE_2_ production in the tendinous tissues. Injection of platelet-poor plasma (PPP) however, did not reduce PGE_2_ levels in the wounded tendons, but the injection of HGF antibody inhibited the effects of PRP and HGF. Further, injection of PRP or HGF also decreased COX-1 and COX-2 proteins. These results indicate that PRP exerts anti-inflammatory effects on injured tendons through HGF. This study provides basic scientific evidence to support the use of PRP to treat injured tendons because PRP can reduce inflammation and thereby reduce the associated pain caused by high levels of PGE_2_.

## Introduction

Tendon injuries are a common condition encountered in orthopaedic surgery and sports medicine practice. In the past few decades, incidences of tendon injuries have increased significantly in both recreational and highly-competitive athletes. Injured tendons tend to heal slowly, particularly when the tendon experiences severe contusion or rupture, which leads to tendon retraction. Furthermore, healing tendons eventually form collagen-rich scar tissue, which impairs tendon function and makes the tendon susceptible to re-injury because the scar tissue has inferior mechanical properties compared to intact tendons [1].

When tissues such as tendons are injured, the first phase of the healing process is inflammation. During this phase, inflammatory agents such as interleukin-1β (IL-1β) are produced by macrophages and other inflammatory cells at the injured site. Additionally, the expression of cyclooxygenase (COX), including the two isoforms COX-1 and COX-2, is upregulated. This upregulation of COX leads to high cellular production of prostaglandin E_2_ (PGE_2_), which is also mediated by PGE_2_ synthase (PGES), including membrane-associated PGE synthase (mPGES) [2]. PGE_2_ is present at high levels in injured tendons and causes vasodilatation [3] and hyperalgesia [4]. It is the target of non-steroid anti-inflammatory drugs (NSAIDs), which are used to reduce pain associated with tissue inflammation. PGE_2_ can also increase the amount of substance P (SP), a major neuro-transmitter of pain sensations [5], which is released in sensory nerves. Therefore, PGE_2_ is considered to be a marker of tendon inflammation [6].

In recent years, platelet-rich plasma (PRP) has been widely adopted in clinics to treat injured tendons [7], as well as many other musculoskeletal tissue injuries [8]. PRP is prepared by simple centrifugation of whole blood to concentrate platelets and simultaneously remove red blood cells. The resultant supernatant is the PRP that contains various growth factors, including platelet derived growth factor (PDGF), transforming growth factor (TGF), fibroblast growth factor (FGF), vascular endothelial growth factor (VEGF), and hepatocyte growth factor (HGF) [9]. These growth factors are involved in the healing of injured tendons [10]–[12] and are able to regulate cellular processes such as chemotaxis, angiogenesis, mitogenesis, differentiation, and metabolism [13]. The rationale behind PRP therapy is that additional platelets will increase the amounts of multiple growth factors released to a localized injury site, which, in turn, will augment the healing process in injured tissues.

In orthopaedic surgery and sports medicine, PRP has been most extensively used in the treatment of ligament and tendon injuries [7]. Many studies have demonstrated favorable outcomes of PRP therapy, such as improved healing of anterior cruciate ligament (ACL) repair in animal models [14]–[16]. PRP treatments have also been reported to improve clinical outcomes in injured tendons, resulting in reduced pain scores along with increased functional scores in elbow tendinosis [17], [18]. In particular, after 1 year of elbow tendinosis treatment with PRP or corticosteroids, 73% of the patients in the PRP treated group had improved pain scores (measured by VAS – Visual Analog Scale and DASH – Disabilities of the Arm, Shoulder and Hand) when compared to ∼50% in the corticosteroid group [19]. In the same patients, after two years without additional intervention, the results remained the same with PRP treated patients showing a declining trend in pain scores, which were significantly lower than the scores before treatment while in the corticosteroid treated group the values were only marginally below baseline scores [20]. These studies suggest that in addition to its stimulatory effects on repair of injured tissues, PRP can reduce tendon inflammation, which is associated with pain. In addition, it is known that HGF, a key growth factor in PRP, has an anti-inflammatory effect on injured organs; for example, it attenuates the renal inflammatory response [21] and protects against lung and liver injuries induced by inflammation [22], [23].

Therefore, in this study we hypothesized that PRP exerts anti-inflammatory effects on injured tendons through HGF. To test this hypothesis, we performed cell culture experiments as an *in vitro* model and a PRP or HGF injection experiment on injured mouse Achilles tendons as an *in vivo* model. To assess whether platelet-poor plasma (PPP) had similar effects to PRP, we further injected PPP into mice. In both *in vitro* and *in vivo* model studies, the expression of COX-1 and COX-2 genes and proteins, the expression of mPGES-1 gene, and the production of PGE_2_, were measured to evaluate cellular and tendon inflammation after treatment with PRP or HGF. The results of this study indicate that PRP has an anti-inflammatory effect, which is mediated by HGF present in PRP preparations.

## Materials and Methods

### Ethics Statement

The University of Pittsburgh IACUC approved all protocols for the use of rabbits for the *in vitro* model studies and mice for the *in vivo* model studies. All surgeries were performed under general anesthesia and efforts were made to minimize suffering to animals.

### Tendon Cell Culture

Tendon cells derived from rabbit patellar tendons of 12 New Zealand white rabbits (4–6 months old females) were used for cell culture experiments. The procedures used to isolate tendon cells were similar to those described previously [24], except that whole tendon cell populations, collectively referred to as tendon cells, were collected and cultured. Briefly, the patellar tendon sheath was removed to obtain the core portion of the tendon. Tendon samples were minced into small pieces and each 100 mg wet tissue sample was digested in 1 ml PBS containing 3 mg collagenase type I and 4 mg dispase at 37°C for 1 hr. After removing the enzymes by centrifugation at 3,500 rpm for 15 min, the cells were cultured in Dulbecco’s modified Eagle’s medium (DMEM; Lonza, Walkersville, MD) supplemented with 20% fetal bovine serum (FBS; Atlanta Biologicals, Lawrenceville, GA), 100 µM 2-mercaptoethanol (Sigma-Aldrich, St. Louise, MO), 100 U/ml penicillin, and 100 µg/ml streptomycin (Atlanta Biologicals, Lawrenceville, GA). The above procedures created a mixed cell population consisting of tendon stem cells (TSCs) and tenocytes, which we defined as tendon cells. In all culture experiments, tendon cells at passage 1 or 2 were used.

### Preparation of PRP and PPP

PRP was prepared from autologous blood of the same animals, which were used for tendon cell culture *in vitro* (rabbit PRP) or *in vivo* (mouse PRP), based on our previously published protocol [25]. This protocol produces PRP with a platelet concentration that is about four times higher than in whole rabbit/mouse blood. Briefly, for *in vitro* experiments, whole rabbit blood (9 ml) was mixed with 1 ml 3.8% sodium citrate (SC) and 10 ml of the blood-SC mixture was centrifuged at 500 g for 30 min. The supernatant containing the concentrated platelets was then collected and counted using Cell-DYN Emerald (Abbott Diagnostics; Lake Forest). To activate platelets, the supernatant was treated with 22 mM CaCl_2_ at 37°C for 1 hr. The resulting supernatant, referred to as PRP, was used immediately or stored at 4°C until used for cell culture experiments.

For the *in vivo* experiments, PRP was obtained as described above from 0.3 ml fresh blood collected from each mouse (n  = 60). The resultant PRP was immediately centrifuged at 10,000 rpm for 30 min at 4°C and the supernatant, referred to as PPP, was transferred to a new tube. After counting the number of platelets contained in the PRP and PPP with Cell-DYN Emerald (Abbott Diagnostics; Lake Forest), both preparations were mixed with 22 mM CaCl_2_, which eventually formed gels and stored at 4°C until used for *in vivo* injection experiments.

### Determination of HGF in Rabbit Whole Blood and PRP

Rabbit whole blood (4.5 ml) was withdrawn from an ear vein of each rabbit and mixed with 0.5 ml 3.8% sodium citrate (SC); 1 ml of blood-SC mixture was used as a whole blood sample, while rest of the 4 ml blood-SC mixture were used for PRP preparations, according to the above protocol. The concentrations of HGF in whole blood and PRP preparations were measured using an ELISA kit (TSZ ELISA, Cat. #RB1842, Framingham, MA). In total, 7 rabbits were used to obtain HGF measurements.

### *In vitro* Experiments

Tendon cells isolated from rabbit patellar tendons at passage 2 were seeded in 6-well plates at a density of 6×10^4^/well and cultured in growth medium (20% FBS-DMEM) for 3 days, at which point cells had reached about 90% confluence. The growth medium was then replaced by serum-free medium (SF; Cat. # S2640; Sigma, St. Louis, MO), and the cells were subjected to the following treatments for 4 hrs. **Group #1:** SF only; **group #2**: SF+IL-1β; **group #3**: SF+PRP+IL-1β; **group #4**: SF+PRP+IL-1β+HGF antibody (AB); **group #5**: SF+PRP; **group #6**: SF+PRP+HGF antibody (AB). A second set of six groups where PRP was replaced with HGF was also maintained. Unless otherwise noted, the following amounts of each component was added to the cultures: 1 ng/ml IL-1β (Cat. # SRP3083; Sigma, St. Louis, MO), a potent inflammatory cytokine, was used to induce cellular inflammation according to a previous study [26]; 10% (v/v) PRP was used to test its effect on tendon cell inflammation; 1 ng/ml of HGF (human recombinant; Cat. # 294HG, R&D Systems, Minneapolis, MN) and 10 ng/ml of HGF antibody (goat anti-human; Cat. # AB-294-NA, R&D systems, Minneapolis, MN) were used wherever needed.

At the end of each treatment, cellular expressions of COX-1, COX-2, and mPGES-1 genes were measured using quantitative RT-PCR (qRT-PCR). In addition, the PGE_2_ production in culture media was measured using an ELISA kit (Cayman Chemical Co., Ann Arbor, MI). COX-1 and COX-2 protein expression levels were also measured using Western blotting (see below).

### Gene Expression Analysis by qRT-PCR

After each treatment, RNA was extracted from cells in each group using the RNeasy Mini Kit with an on-column DNase I digest (Qiagen, Valencia, CA). First-strand cDNA was synthesized from 1 µg total RNA in a 20 µl reaction by reverse transcription using SuperScript II (Invitrogen, Grand Island, NY). The following conditions for cDNA synthesis were applied: 65°C for 5 min and cooling at 4°C for 1 min, then 42°C for 50 min and 72°C for 15 min. Next, qRT-PCR was performed using QuantiTect SYBR Green PCR Kit (QIAGEN, Valencia, CA). In a 25 µl PCR reaction mixture, 2 µl cDNA (total 100 ng RNA) were amplified in a Chromo 4 Detector (MJ Research, St. Bruno, Quebec, Canada) by incubation at 94°C for 5 min, followed by 30 to 40 cycles of a three temperature program consisting of 1 min at 94°C, 40 seconds at 57°C, and 50 seconds at 72°C. The PCR reaction was terminated after a 10 min extension at 70°C. Rabbit-specific primers for COX-1, COX-2, mPGES-1, and glyceraldehyde 3-phosphate dehydrogenase (GAPDH), which was used as an internal control, were adopted from previous studies (**Table 1**) [27], [28] and synthesized by Invitrogen. A no template control (NTC) without cDNA was included for each primer pair and each reaction had at least three replications. All parameters including PCR efficiency, primer calibration and product specificity were verified using standard qRT-PCR protocols. Relative expression levels of each gene was calculated from the formula 2^−ΔΔCT^, where ΔΔCT = (CT_target_ – CT_GAPDH_)_treated_ – (CT_target_ – CT_GAPDH_)_control_. CT represents the cycle threshold of each cDNA sample.

**Table 1:** Genes and primer sequences used in qRT-PCR.

Genes	Primer sequences (Forward/Reverse)	References
COX-1	5′-TAGTGGACGCCTTCTCTCGC-3′ 5′-CCGCTGCCATCTCTGTCTCT-3′	[27]
COX-2	5′-GGTGGAGATGATCTACCCGC-3′ 5′-GGTTGAAAAGCAGCTCTGGG-3′	[27]
mPGES-1	5′-GCAGCGCACTGCTGGTTCTGAAGA-3′ 5′-AGACCAGGCCCAGGAAGAGGAAA-3′	[28]
GAPDH	5′-AGGGTCATCATCTCAGCCCC-3′ 5′-ATGCCTGCTTCACCACCTTC-3′	[27]

### Western Blotting to Measure COX-1 and COX-2 Expression

At the end of each treatment, protein was first extracted from cells in each group using the M-PER mammalian protein extraction reagent (Thermo Scientific; Cat. #78505; Rockford, IL), and then separated on 12% sodium dodecyl sulfate-polyacrylamide gel electrophoresis (SDS-PAGE) gel. The proteins were then transferred onto Nitrocellulose membrane (Invitrogen; Cat. #LC-2000; Carlsbad, CA) using a semi-dry blotting apparatus (Bio-Rad; Hercules, CA) for 60 min at 2.0 mA/cm^2^. COX-1 and COX-2 proteins were then detected using the following antibodies: goat anti-COX-1 polyclonal antibody (1∶500 room temperature for 3 hrs; Thermo Scientific; Cat. #PA1-85118; Rockford, IL) and goat anti-COX-2 polyclonal antibody (1∶500 at room temperature for 3 hrs; Abcam; Cat. # ab35995; Cambridge; MA) respectively. Mouse anti-GAPDH antibody was used as a loading control (1∶500 at room temperature for 3 hrs; Abcam; Cat. #ab9484; Cambridge; MA) to detect GAPDH. Then, peroxidase-conjugated mouse anti-goat IgG antibody (1∶1000 at room temperature for 2 hrs; Santa Cruz; Cat: #sc-2354; Dallas, TX 75220) was used as the secondary antibody to detect COX-1 and COX-2, and goat anti-mouse IgG-HRP antibody (1∶1000 at room temperature for 2 hrs; Santa Cruz; Cat: #sc-2055; Dallas, TX 75220) was used for GAPDH. Finally, the ECL Western Blotting Analysis System (GE Healthcare, Cat. #RPN 2108, Buckinghamshire, HP, UK) was used to detect the protein bands, which were visualized after exposure to X-ray films.

### Immunostaining to Detect COX-1 and COX-2 Expression

Tendon cells at passage 2 were seeded in 12-well plates at a density of 3×10^4^/well and treated with IL-1β, PRP, HGF, and/or HGF antibody for 4 hrs before determining COX-1 and COX-2 expressions. After each treatment, COX-1 expression was tested using goat anti-COX-1 (1∶300) polyclonal antibody (Thermo Scientific; Cat. #PA1-85118; Rockford, IL) and goat anti-COX-2 (1∶300) polyclonal antibody (Abcam; Cat. # ab35995; Cambridge; MA) at room temperature for 2 hrs followed by Cy3-conjugated donkey anti-goat IgG (1∶500) at room temperature for 1 hr as secondary antibody to detect both COX-1, and COX-2 expression. The stained cells were examined using fluorescence microscopy.

### *In vivo* Injection Experiments

For *in vivo* injection experiments, mice instead of rabbits were used. PRP and PPP from mice were prepared as described under ‘Preparation of PRP and PPP’. Under anesthesia, a wound with a 1 mm diameter was created on the Achilles tendon of each mouse using a biopsy punch (Miltex, Inc., York, PA). The wounded mice were divided into seven groups and the following injections were given to the mice immediately after wounding: 20 mice were given a 10 µl saline injection in each wound (wound only group); 20 mice were injected with 10 µl PRP in each wound (PRP group); 20 mice were injected with 10 µl PPP (PPP group); 20 mice were given an injection of 3 ng HGF in 10 µl of an engineered tendon matrix (ETM) prepared from rabbit patellar tendon according to the published protocol [29] in each wound (HGF group); 20 mice were given an injection of 3 ng HGF and 10 ng HGF antibody (AB) in 10 µl of ETM (HGF+AB group); 20 mice were injected with 10 µl ETM (ETM), and 20 mice were injected with 10 µl PRP and 10 ng HGF antibody (AB) in 10 µl saline into each wound (PRP+AB group). In these experiments PRP and PPP were injected directly because both form gels in the wound area. However, since HGF and anti-HGF antibody remain as liquids, when injected individually or together, they were mixed with a carrier such as ETM that forms a matrix after injection thereby limiting the molecules to the wound area. After treatment, all mice were allowed free cage activities. Four mice in each group were sacrificed on days 0, 1, 3, 5, and 12 post-treatment, and the Achilles tendons (two from the hind limbs of each mouse) were harvested. Day 0 tendons were removed from mice immediately after injections. PGE_2_ levels in tendon tissues were measured using an ELISA kit (Cat. # 514010, Cayman Chemical Co, Ann Arbor, MI), and COX-1 and COX-2 expressions were measured by immunostaining of frozen tissue sections. For immunostaining, the tissue samples were immersed into frozen section medium (Neg 50; Richard-Allan Scientific; Kalamazoo, MI) in pre-labeled base molds and were quickly frozen by placing in 2-methylbutane chilled with liquid nitrogen. The frozen tissue blocks were then placed on dry ice and subsequently stored in a deep freezer (−80°C) until they were used for histological and immunohistochemical analyses.

### COX-1 and COX-2 Expression in Tissue Sections Determined by Immunohistochemistry

Each tissue was cut into 8 µm thick sections and fixed in 4% paraformaldehyde for 15 min. The fixed sections were stained with goat anti-COX-1 (1∶300) polyclonal antibody (Thermo Scientific; Cat. #PA1-85118; Rockford, IL) at room temperature for 2 hrs, goat anti-COX-2 (1∶300) polyclonal antibody (Abcam; Cat. # ab35995; Cambridge; MA) at room temperature for 2 hrs for primary binding, and FITC-conjugated rabbit anti-goat IgG for COX-1 (1∶500) at room temperature for 1 hr, or Cy-3 conjugated donkey anti-goat IgG antibody for COX-2 (1∶500) at room temperature for 1 hr for secondary binding and detection. Stained cells were examined using fluorescence microscopy.

### Measurement of PGE_2_ in Tendon Tissues

The procedure for PGE_2_ measurement is as follows: Mouse Achilles tendon samples were weighed, minced, placed in buffer (100 µl buffer for each 1 mg tissue) provided by the ELISA kit manufacturer (Cayman Chemical Co., Ann Arbor, MI), and homogenized. The tissue samples were centrifuged at 3,000 g for 30 min at 4°C, and the supernatant was collected to measure PGE_2_ using an ELISA kit according to manufacturers’ instructions. The measured values were normalized with respect to tissue weight.

### Data Analysis

For each experimental condition, at least three replicates were used. The findings presented in the results are representative of these three replicates. All gene expression data gathered from *in vitro* experiments were normalized to the serum-free group (SF), which was the control. A two-tailed student *t*-test was used for statistical analysis. A P-value less than 0.05 was considered to indicate a statistically-significant difference between two groups.

## Results

### The Concentration of HGF in Whole Blood and PRP

**Figure 1:**
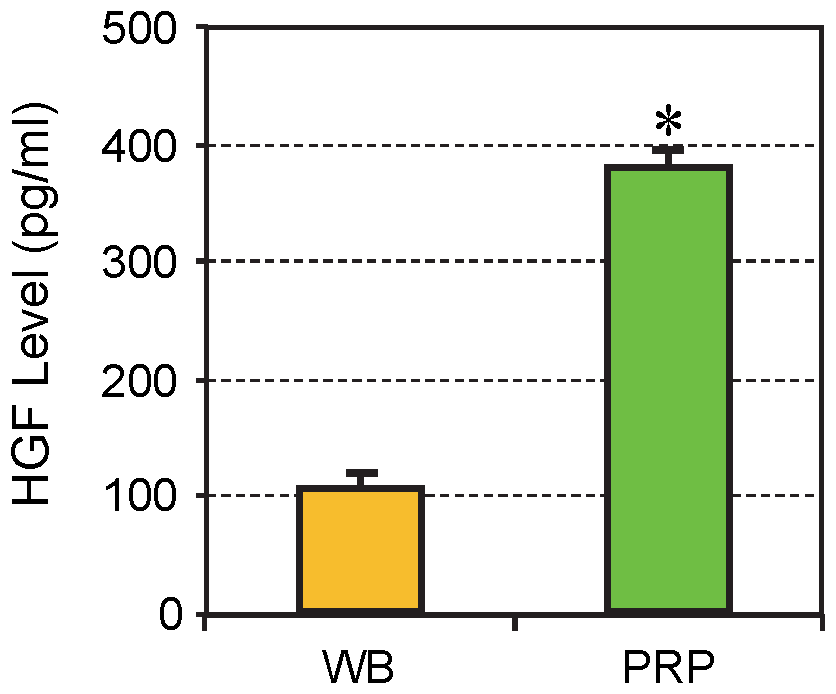
HGF levels in rabbit whole blood and PRP preparations. The concentration of HGF in PRP was over 3.5 times the concentration found in rabbit whole blood (WB). The data are expressed as mean ± SD, n  = 7.

PRP preparations used in tendon cell culture experiments contained on an average, more than 3.5 times the level of HGF in rabbit whole blood (**Figure 1**).

### The Effects of PRP and HGF Treatments *in vitro*

#### COX-1 and COX-2 mRNA expression

**Figure 2:**
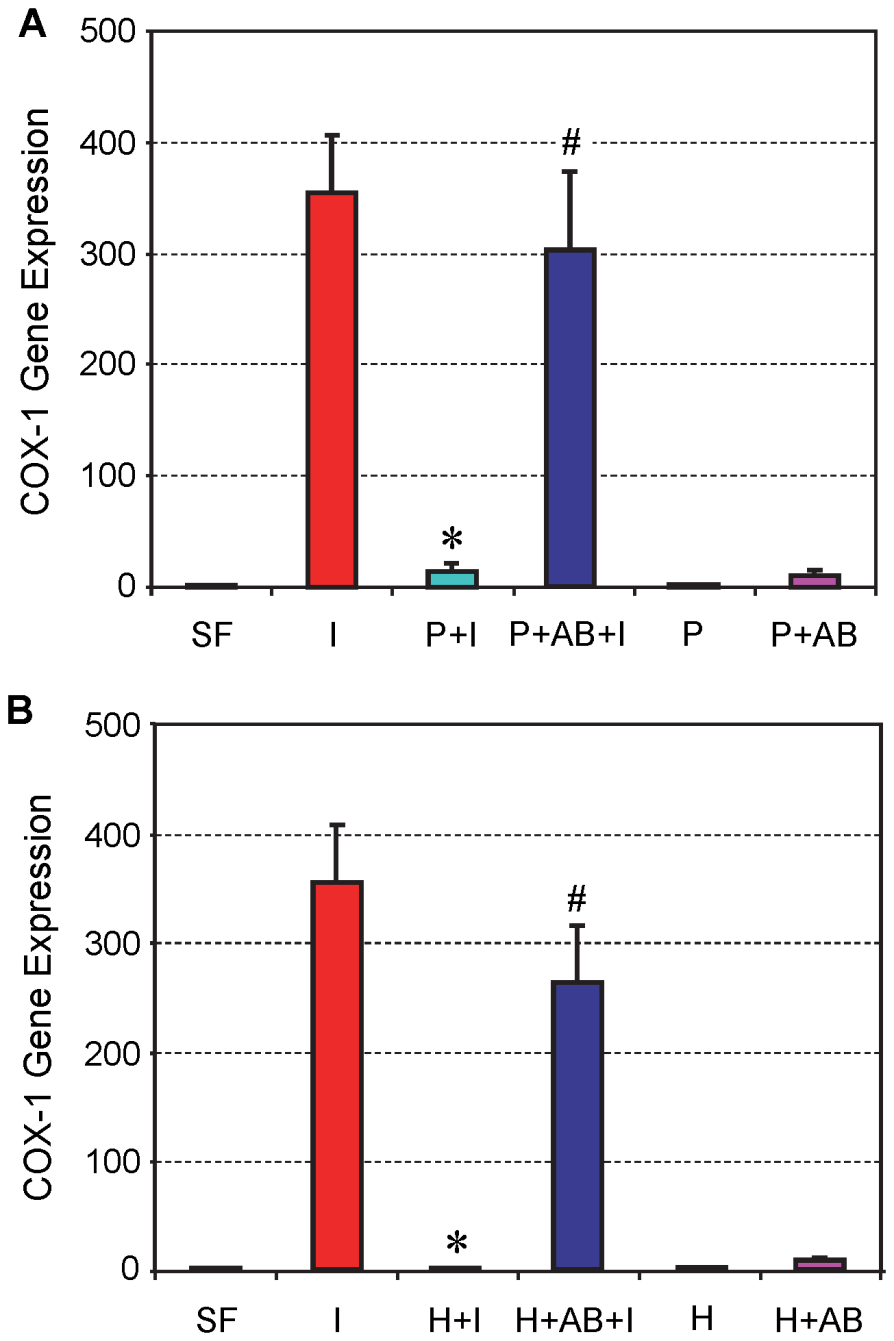
The effect of PRP (A) and HGF (B) treatment on COX-1 expression in tendon cell culture. Tendon cells were treated with IL-1β, which resulted in high levels of COX-1 expression; however, the addition of PRP (**A**) or HGF (**B**) together with IL-1β to the cell culture markedly reduced the level of COX-1 expression. Also, PRP or HGF alone did not alter COX-1 expression. Note that *P<0.05 is with respect to IL-1β treatment (**I**); and ^#^P<0.05 is with respect to the PRP+IL-1β (**P+I**) group in **A** and the HGF+IL-1β (**H+I**) group in **B**. The data are expressed as mean ± SD, n  = 4. **SF:** serum free medium; **I:** IL-1β; **P+I:** PRP+IL-1β; **P+AB+I:** PRP+HGF antibody+IL-1β; **P:** PRP; **P+AB:** PRP+HGF antibody; **H+I:** HGF+ IL-1β; **H+AB+I:** HGF+HGF antibody+IL-1β, **H:** HGF; and **H+AB:** HGF+HGF antibody. Concentrations of the reagents used were: IL-1β, 1 ng/ml; PRP, 10% (v/v); HGF, 1 ng/ml; HGF antibody, 10 ng/ml. For SD values that are barely visible, the variation in gene expression values was less than 5 (control group, SF is 1).

Tendon cell cultures were treated with 1 ng/ml of IL-1β to induce cellular inflammation. After the addition of IL-1β, COX-1 gene expression in tendon cells was highly elevated (I) compared to non-treated cells (SF) (**Figure 2A**). However, once 10% PRP was added with IL-1β (P+I), COX-1 expression was almost completely suppressed. Addition of HGF antibody to the PRP/IL-1β mix (P+AB+I) abolished the suppressive effects of PRP and recovered about 90% of COX-1 levels when compared to induction with IL-1β alone. This indicates an interaction between PRP and HGF antibody, and their influence on COX-1 expression. Additionally, neither PRP alone (P) nor PRP plus HGF antibody (P+AB) affected COX-1 expression (**Figure 2A**).

In a parallel culture experiment, the addition of HGF to tendon cell culture that had been treated with IL-1β (H+I) resulted in suppressive effects similar to those produced by PRP (**Figure 2B**); that is, the application of HGF (1 ng/ml) nearly blocked IL-1β induced COX-1 expression in tendon cells. The addition of HGF antibody (10 ng/ml) along with HGF (1 ng/ml) greatly reduced HGF’s suppressive effects on IL-1β induced COX-1 expression (H+AB+I). However, such a combined use of HGF antibody with HGF only partially (∼70%) restored the COX-1 expression levels induced by IL-1β treatment, suggesting that a higher dose of HGF antibody may be needed to completely block the suppressive effects of HGF on COX-1 expression.

**Figure 3:**
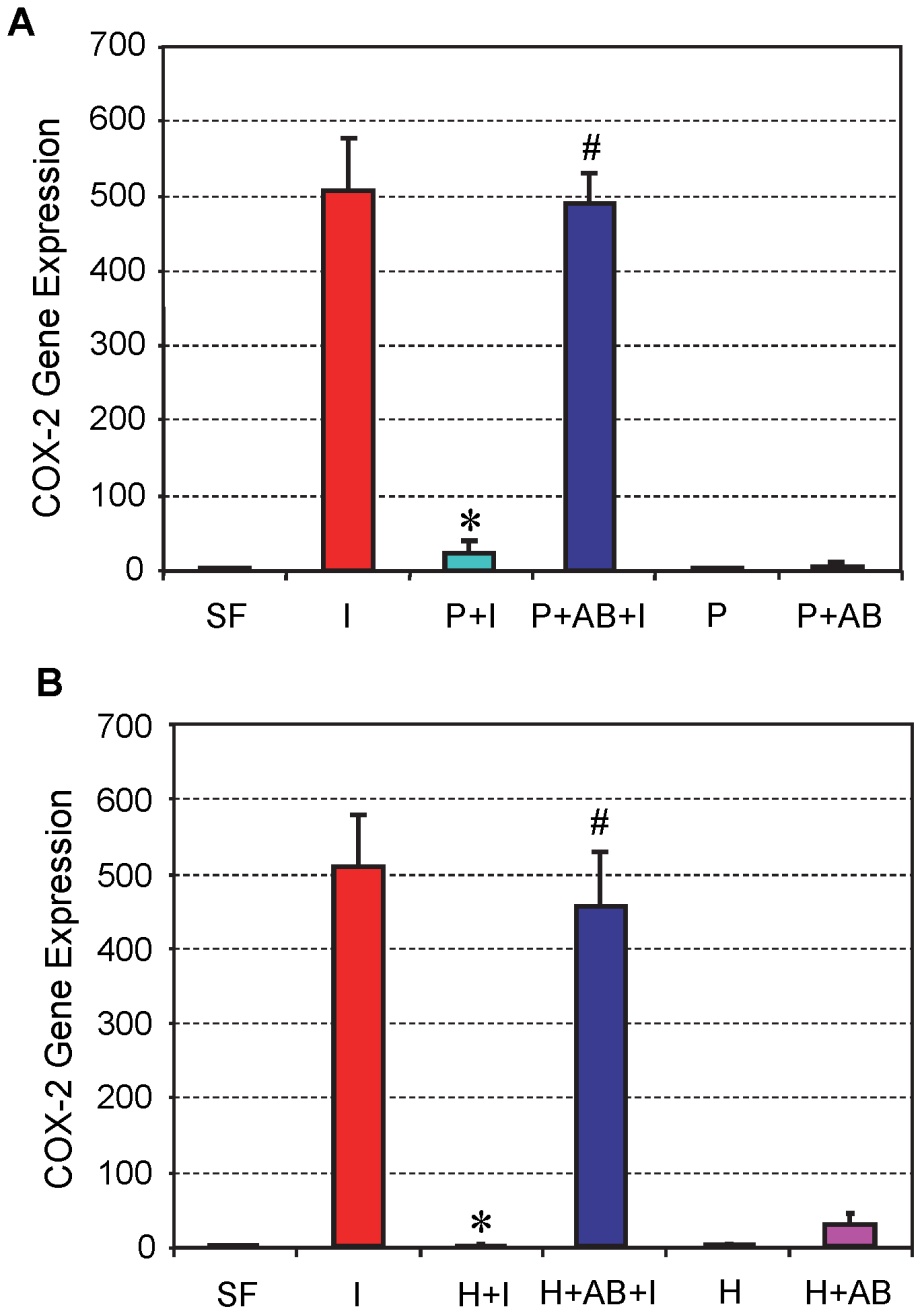
The effects of PRP (A) and HGF (B) treatment on COX-2 gene expression in tendon cell culture. Both PRP and HGF treatments nearly abolished COX-2 expression, which was induced by IL-1β treatment of tendon cells. Note that *P<0.05 is with respect to IL-1β treatment condition (**I**); and ^#^P<0.05 is with respect to the PRP+IL-1β (**P+I**) group in **A** and the HGF+IL-1β (**H+I**) group in **B**. The data are expressed as mean ± SD, n  = 4. Label abbreviations and concentrations of all agents (IL-1β, PRP, HGF, and HGF antibody) are identical to legends in **Figure 2**. For SD values that are barely visible, the variation in gene expression values was less than 5 (control group, SF is 1).

The level of COX-2 expression in tendon cell culture was similar to COX-1. Specifically, the same amount of IL-1β (1 ng/ml) treatment caused a drastic increase in COX-2 gene expression (I), but addition of 10% PRP to cell cultures almost abolished COX-2 expression (P+I). The addition of HGF antibody (10 ng/ml) with PRP (P+AB+I) nearly restored the level of COX-2 expression to control levels induced by IL-1β treatment (**Figure 3A**). Likewise, treatment of tendon cell cultures with both HGF and HGF antibody (H+AB+I) yielded results similar to those produced by treatment with PRP and HGF antibody (**Figure 3B**), indicating an interaction between PRP and HGF on the IL-1β induced expression levels of COX-2.

#### mPGES-1 gene expression

**Figure 4:**
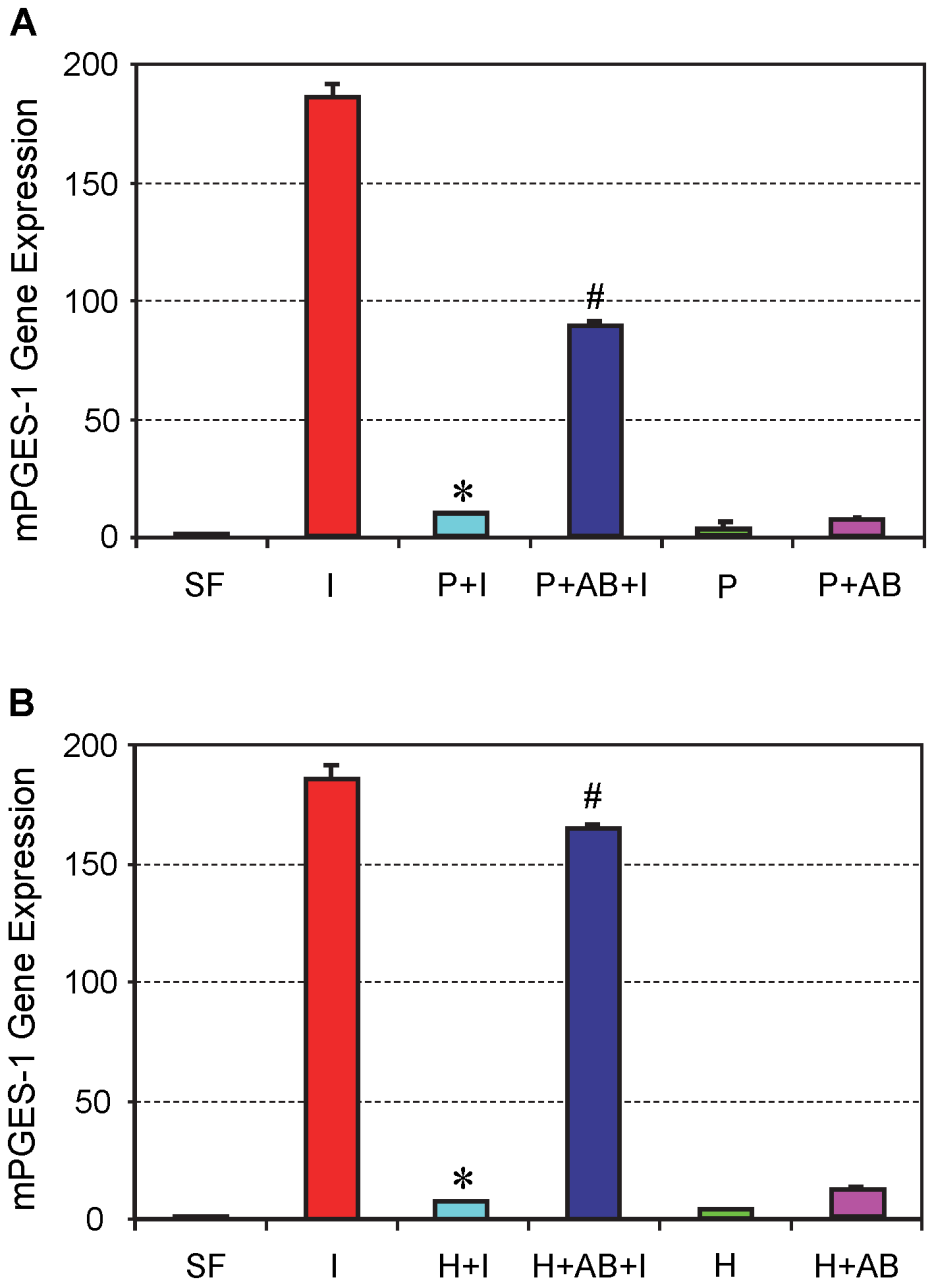
The effects of PRP (A) and HGF (B) treatment on mPGES-1 gene expression in tendon cell culture. IL-1β treatment increased mPGES-1 expression (**I**), but its effect was almost completely suppressed by PRP (**A**) or HGF (**B**) treatment. Note that *P<0.05 is with respect to IL-1β treatment condition (**I**); and ^#^P<0.05 is with respect to the PRP+IL-1β (**P+I**) group in **A** and the HGF+IL-1β (**H+I**) group in **B**. The data are expressed as mean ± SD, n  = 4. Label abbreviations and concentrations of all agents (IL-1β, PRP, HGF, and HGF antibody) are identical to legends in **Figure 2**. For SD values that are barely visible, the variation in gene expression values was less than 5 (control group, SF is 1).

In addition, PRP treatment suppressed mPGES-1 expression induced by IL-1β treatment in tendon cell cultures (P+I), whereas the addition of HGF antibody reduced the suppressive effects of PRP (P+AB+I) (**Figure 4A**). HGF treatment produced results similar to PRP treatment, although HGF antibody together with HGF “restored” higher percentage of mPGES-1 expression than with PRP (∼50% restoration with PRP vs. >90% with HGF) (**Figure 4B**).

#### PGE_2_ production

**Figure 5:**
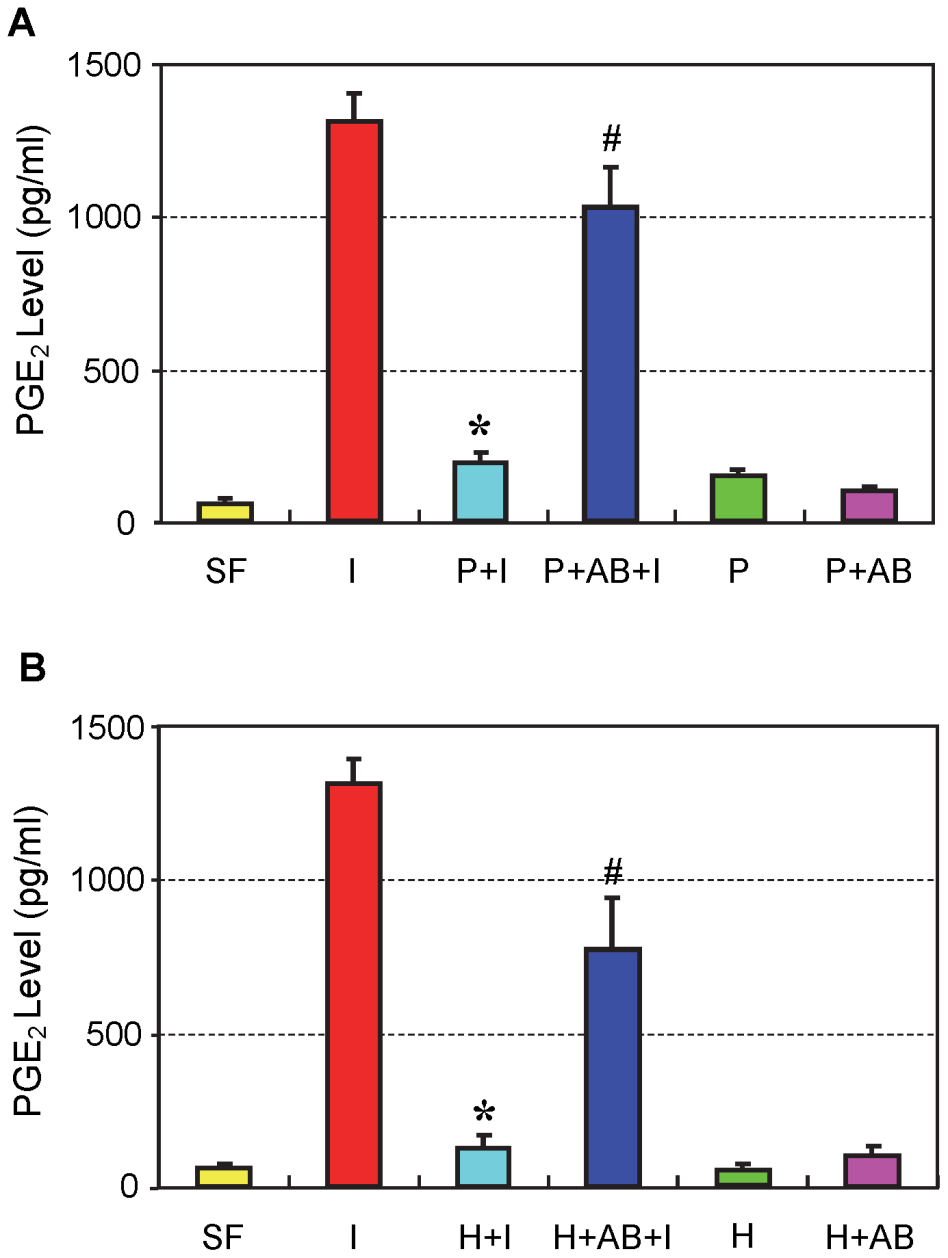
The effects of PRP (A) and HGF (B) treatments on PGE_2_ production in tendon cell culture. IL-1β treatment (**I**) induced high levels of PGE_2_ production in cells compared to levels induced by control conditions (**SF**); however, these increases in PGE_2_ were markedly reduced by PRP or HGF treatment. Note that *P<0.05 is with respect to IL-1β treatment condition (**I**); and ^#^P<0.05 is with respect to the PRP+IL-1β (**P+I**) group in **A** and the HGF+IL-1β (**H+I**) group in **B**. The data are expressed as mean ± SD, n  = 4. Label abbreviations and concentrations of all agents (IL-1β, PRP, HGF, and HGF antibody) are identical to legends in **Figure 2**. For SD values that are barely visible, the variation in gene expression values was less than 5 (control group, SF is 1).

PRP treatment nearly blocked the cellular production of PGE_2_ that was elevated by IL-1β treatment of tendon cell cultures (**Figure 5A**). HGF treatment produced results similar to those with PRP treatment (**Figure 5B**). The addition of HGF antibodies to PRP or HGF in cell cultures partially (∼60–80%) restored PGE_2_ production levels compared to IL-1β treated tendon cells.

#### COX-1 and COX-2 protein expression in vitro by western blot

**Figure 6:**
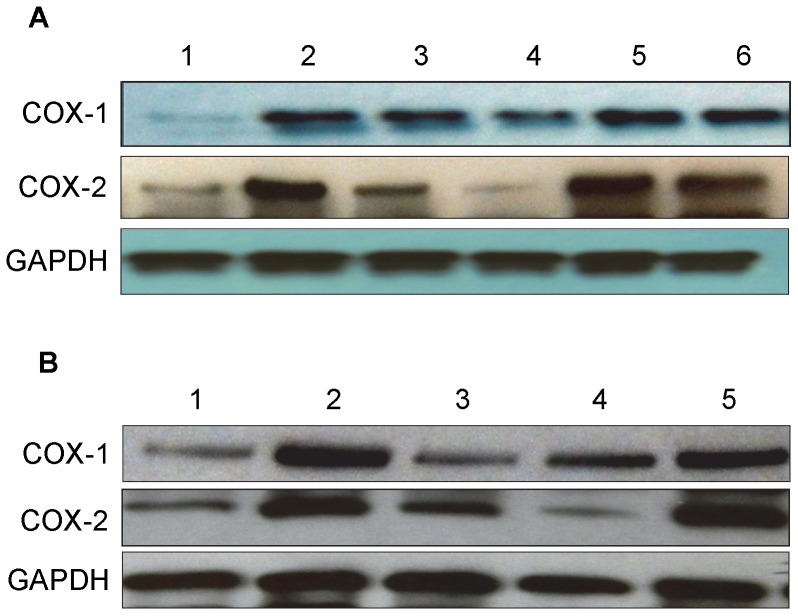
The effects of PRP (A) and HGF (B) treatment on COX-1 and COX-2 protein expression in tendon cell culture. Total proteins extracted from tendon cells after treatments were separated on 12% SDS-PAGE and transferred onto nitrocellulose membrane to detect COX-1 and COX-2 proteins using goat anti-COX-1 and goat anti-COX-2 polyclonal antibodies, respectively, and peroxidase-conjugated rabbit anti-goat IgG antibody as secondary antibody. **A.** PRP treatment. Lane 1: serum free medium (SF); lane 2: IL-1β (1 ng/ml); lane 3: PRP (2%)+IL-1β (1 ng/ml); lane 4: PRP (10%)+ IL-1β (1 ng/ml); lane 5: PRP (2%)+ IL-1β (1 ng/ml)+HGF antibody (10 ng/ml); lane 6: PRP (10%)+IL-1β (1 ng/ml)+HGF antibody (10 ng/ml). Notice that 10% PRP reduced COX-1 expression, but almost completely inhibited COX-2 expression (lane 4 vs. lane 2). **B.** HGF treatment. Lane 1: SF; lane 2: IL-1β (1 ng/ml); lane 3: HGF (1 ng/ml)+IL-1β (1 ng/ml); lane 4: HGF (1 ng/ml); and lane 5: HGF (1 ng/ml)+IL-1β (1 ng/ml)+HGF antibody (10 ng/ml). Also notice that HGF at 1 ng/ml markedly reduced both COX-1 and COX-2 expression (lane 3 vs. lane 2).

The addition of IL-1β to tendon cell cultures increased both COX-1 and COX-2 protein expression levels (lane 2), compared to the levels in the control group (lane 1). PRP treatment decreased the effect of IL-1β on COX-1 and COX-2 expressions in an apparent dose-dependent manner (lanes 3, 4). Moreover, PRP’s suppressive effect was nullified by the addition of HGF antibody (lanes 5, 6) (**Figure 6A**). HGF treatment also inhibited the effects of IL-1β on COX-1 and COX-2 expressions and HGF antibody decreased these inhibitory effects (**Figure 6B**).

#### The effects of PRP and HGF on COX-1 and COX-2 protein levels by immunostaining

**Figure 7:**
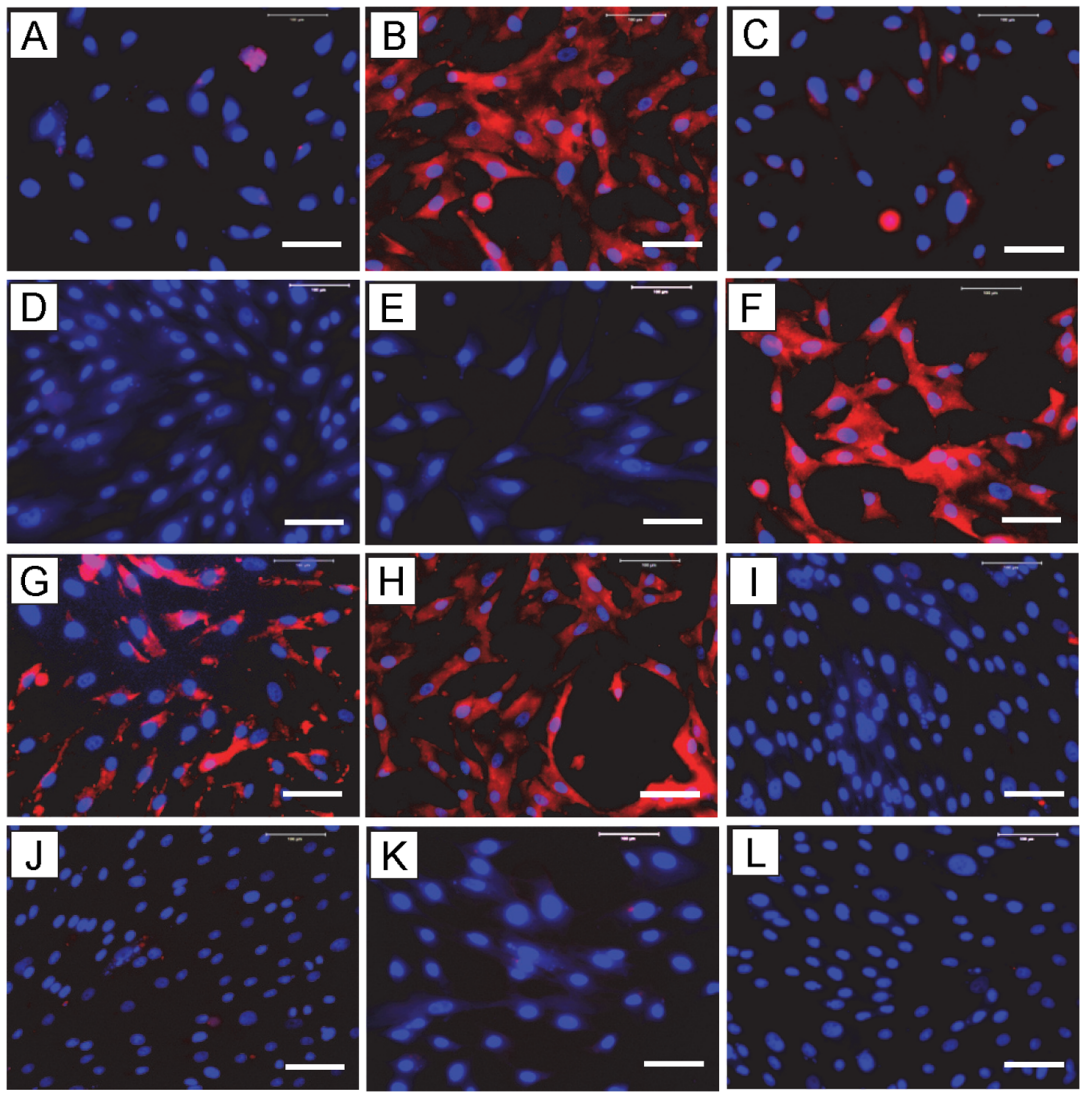
The effects of PRP or HGF treatment on COX-1 protein expression in tendon cell culture by immunostaining. Tendon cells in culture were treated with IL-1β, PRP, HGF, and/or HGF antibody (AB) to determine COX-1 expression using goat anti-COX-1 antibody and Cy-3 conjugated donkey anti-goat IgG antibody. Red represents COX-1 protein; and blue represents nuclei stained with Hoechst 33342. IL-1β treatment induced a high level of COX-1 expression in tendon cells compared to the control conditions (**A**); however, such an increase in COX-1 was markedly reduced by PRP or HGF treatments. The addition of HGF antibody to tendon cell cultures decreased the reduction by PRP or HGF treatments. **A:** SF (control conditions); **B:** IL-1β (1 ng/ml); **C:** 2%PRP+ IL-1β; **D:** 10%PRP+IL-1β; **E:** HGF+IL-1β; **F:** 2%PRP+IL-1β+AB; **G:** 10%PRP+IL-1β+AB; **H:** HGF+IL-1β+AB; **I:** 2%PRP; **J:** 10%PRP; **K:** HGF (1 ng/ml) and **L:** HGF+AB (10 ng/ml). Bar: 100 µm.

Immunostaining showed that when compared to the control (**Figure 7A**) nearly all tendon cells treated with IL-1β were positively stained for COX-1 (**Figure 7B**). The cellular inflammatory response, which is the effect of IL-1β on COX-1 protein expression in this study, was inhibited ∼50% by 2% PRP (**Figure 7C**), but was completely inhibited by 10% PRP (**Figure 7D**) and HGF (1 ng/ml) (**Figure 7E**). On the other hand, the addition of 10 ng/ml of HGF antibody to tendon cell cultures blocked such suppressive effects of HGF and PRP completely (**Figure 7F, G, H**). Tendon cells treated only with 2% PRP (**Figure 7I**), 10% PRP (**Figure 7J**), HGF (1 ng/ml) (**Figure 7K**), or HGF+HGF antibody (AB, 10 ng/ml) (**Figure 7L**) without IL-1β did not show alterations in the levels of COX-1 but remained similar to the control (**Figure 7A**).

**Figure 8:**
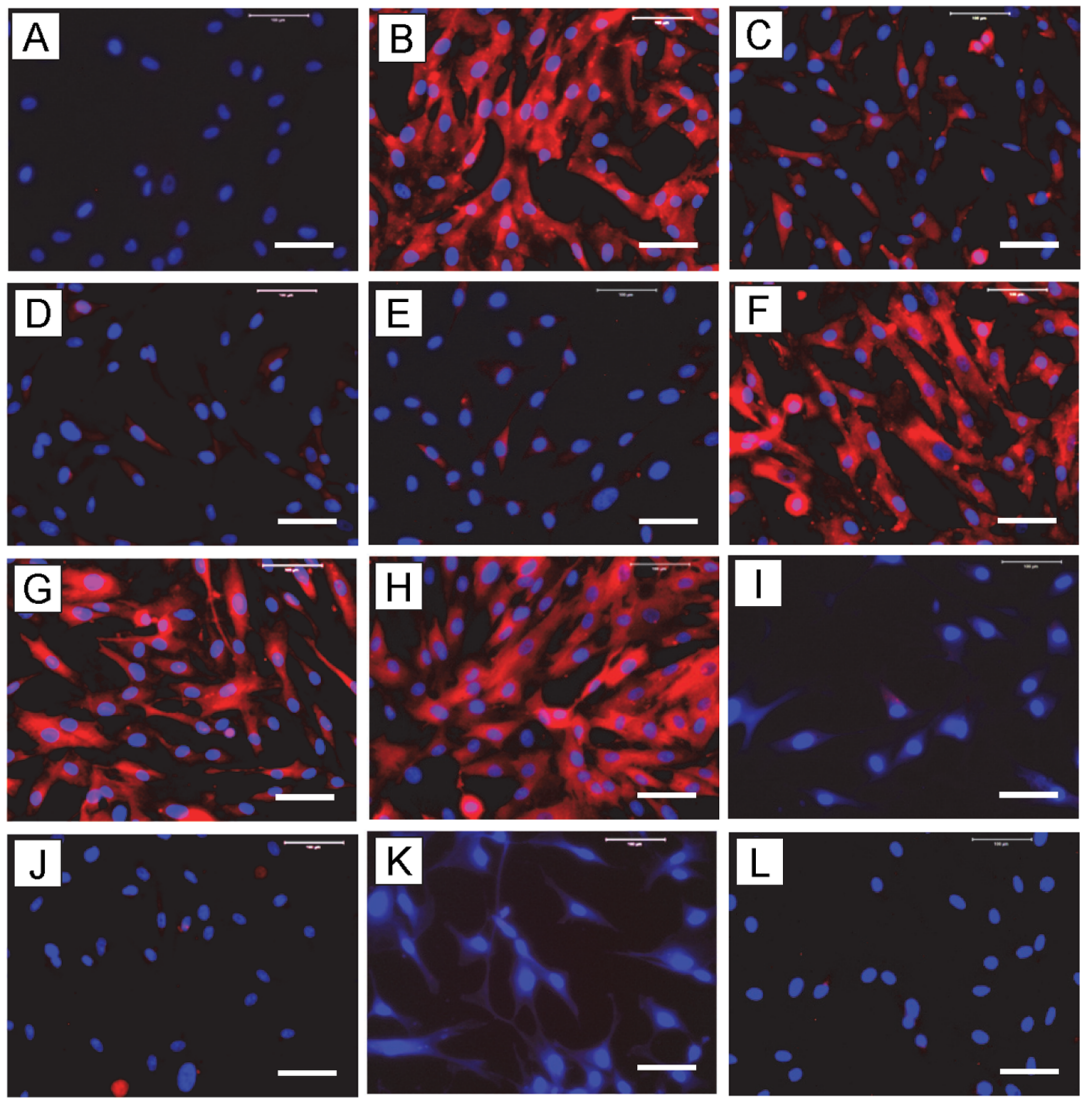
The effects of PRP or HGF treatment on COX-2 protein expression in tendon cell culture by immunostaining. Tendon cells in culture were treated with IL-1β, PRP, HGF, and/or HGF antibody (AB) to determine COX-2 expression using goat anti-COX-2 antibody and Cy-3 conjugated donkey anti-goat IgG antibody. Red represents COX-2 protein; and blue represents nuclei stained with Hoechst 33342. Similar to COX-1 results (**Figure 7**), PRP or HGF treatments markedly reduced COX-2 expression, and the combined use of HGF antibody with PRP or HGF decreased the reduction effects produced by either PRP or HGF treatment alone. Label abbreviations (A to L) are the same as in **Figure 7**. Bar: 100 µm.

Similarly, COX-2 expression induced by IL-1β (**Figure 8B**) was more than 25% inhibited by 2% PRP (**Figure 8C**), 50% inhibited by 10% PRP (**Figure 8D**), and more than 60% by 1 ng/ml HGF (**Figure 8E**). The HGF antibody completely abolished the suppressive effects of PRP and HGF on COX-2 (**Figure 8F, G, H**). Similar to COX-1, the expression of COX-2 remained unchanged when treated only with 2% PRP (**Figure 8I**), 10% PRP (**Figure 8J**), HGF (1 ng/ml) (**Figure 8K**), or HGF+HGF antibody (AB, 10 ng/ml) (**Figure 8L** **)** without IL-1β.

### The Effects of PRP and PPP Treatments *in vivo*

**Figure 9:**
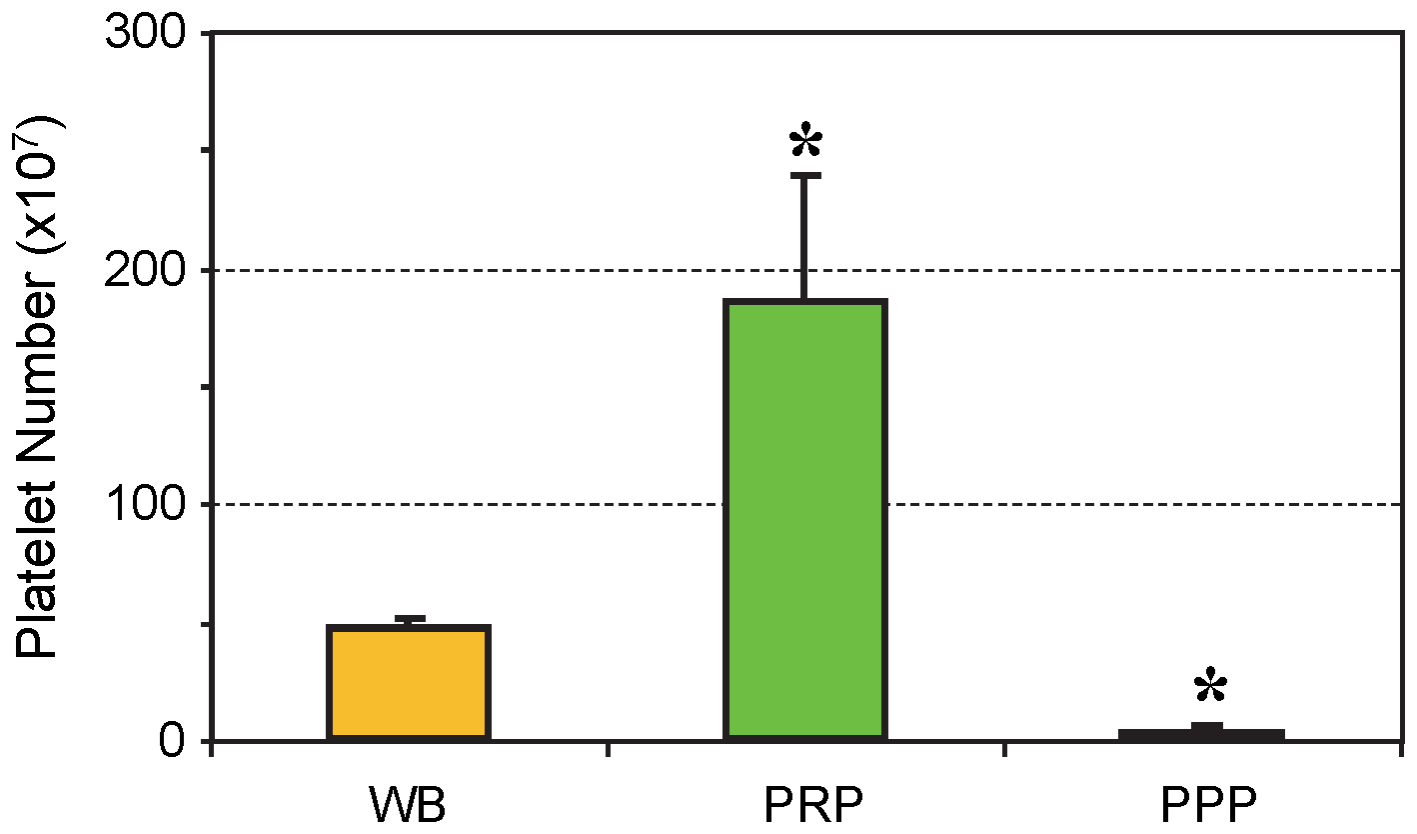
Platelet numbers in mouse whole blood, PRP, and PPP preparations used for the *in vivo* experiments. On average, the number of platelets in PRP preparations was about four times higher than in mouse whole blood (WB), whereas the number was 60 times higher in PRP than in PPP preparations (*P<0.05 is with respect to WB). The data are expressed as mean ± SD, and n  = 8.

The number of platelets in PRP used for *in* *vivo* injections was about four times higher than the number of platelets in mouse whole blood and about 60 times higher than the number of platelets in PPP, which was used for a control group (**Figure 9**).

#### PGE_2_ production *in vivo*

**Figure 10:**
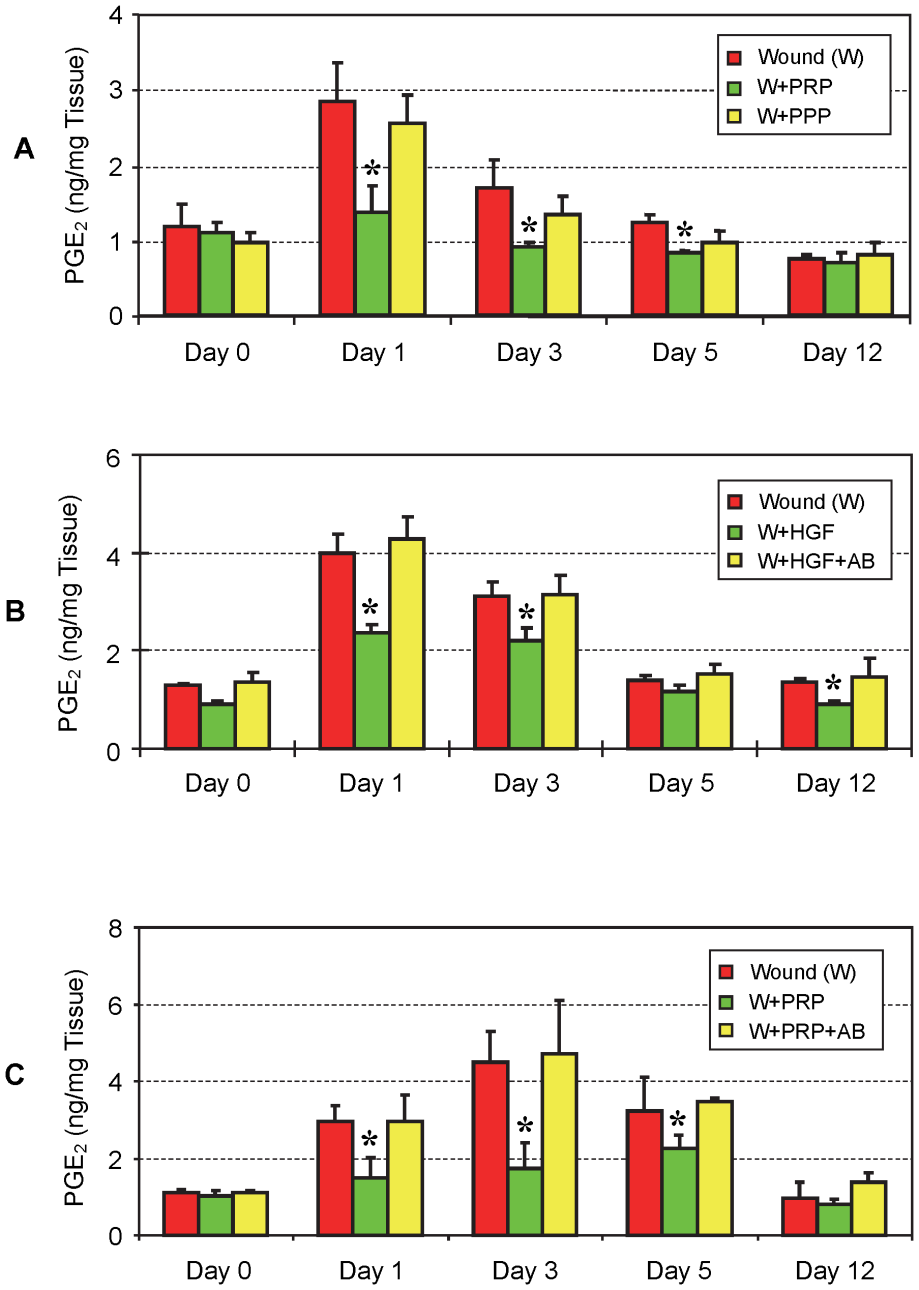
The effects of PRP, PPP, HGF and HGF antibody on PGE_2_ production in wounded mouse Achilles tendons *in vivo*. **A.** PRP and PPP injections. Injections with PRP significantly reduced PGE_2_ levels in tendons on days 1, 3, and 5 post-treatment. However, on day 12, PRP’s effect on PGE_2_ production in tendon tissues was little, if any. On day 0, little difference was observed in PGE_2_ levels between PRP-treated and non-treated tendons. However, PPP treatment did not significantly decrease PGE_2_ levels in the tendon tissues at all time points post-treatment (P>0.05). However, a small suppression effect on days 1, 3, and 5 likely occurred, as PPP-treated tendons had consistently lower PGE_2_ levels than control tendons injected with saline. **B.** HGF injection. Injection with HGF decreased PGE_2_ production in wounded Achilles tendons, producing results similar to PRP injections except on day 12, when HGF injection still significantly decreased PGE_2_ production (*P<0.01 is with respect to control tendons given saline injections). Addition of HGF+HGF antibody (AB) largely negated the suppressive effect of HGF. **C.** PGE_2_ production in mouse tendons injected with PRP and HGF antibody. Injection of PRP along with HGF antibody into wounded Achilles tendons restored PGE_2_ levels to normal on all days 1, 3, 5 and 12. The data in **Figure 10A, B, C** are expressed as mean ± SD, and n  = 4 (4 mice).

PRP injections did not significantly reduce PGE_2_ levels immediately after injection on day 0, but they significantly decreased PGE_2_ levels in wounded mouse Achilles tendons on days 1, 3, and 5 post-treatment. On day 12, PGE_2_ levels in both PRP injected and un-injected groups were lower than on day 0 with no apparent effect of the PRP treatment remaining (**Figure 10A**). On the other hand, PPP injections did not significantly reduce the levels of PGE_2_ in mouse Achilles tendons at any of the above time points (P>0.05). However, as revealed by a trend analysis, PPP slightly reduced PGE_2_ production in the mouse tendons on days 1, 3, and 5 (**Figure 10A**). This is more likely due to the low abundance of platelets in PPP (**Figure 9**). Furthermore, HGF injections reduced PGE_2_ levels similar to PRP injections. Specifically, HGF significantly decreased PGE_2_ levels on days 1, 3 and 12, albeit to varying degrees (**Figure 10B**). On day 5, a slight reduction in PGE_2_ levels was observed which was not statistically significant. Note that on day 0 the treatment groups exhibited a transient response to HGF treatment, as they had lower PGE_2_ levels than the control group. This was likely due to the lag time (∼1 hr) from injection of HGF to harvesting and processing of mouse tendinous tissues for PGE_2_ measurement. Further, the injection of HGF antibody into wounded mouse Achilles tendons, completely inhibited the HGF effect and PGE_2_ production was similar to the control (wounded only group) (**Figure 10B**). Addition of HGF antibody into wounded Achilles, also blocked the suppressive effect exerted by PRP, and PGE_2_ production was much higher than the group injected with PRP only (**Figure 10C**). In these experiments, the results from saline injections (wound only group – red columns) were similar to those from ETM injections; therefore, the results from the saline injected group only are presented here as the control.

#### COX-1 and COX-2 protein expression *in vivo*

**Figure 11:**
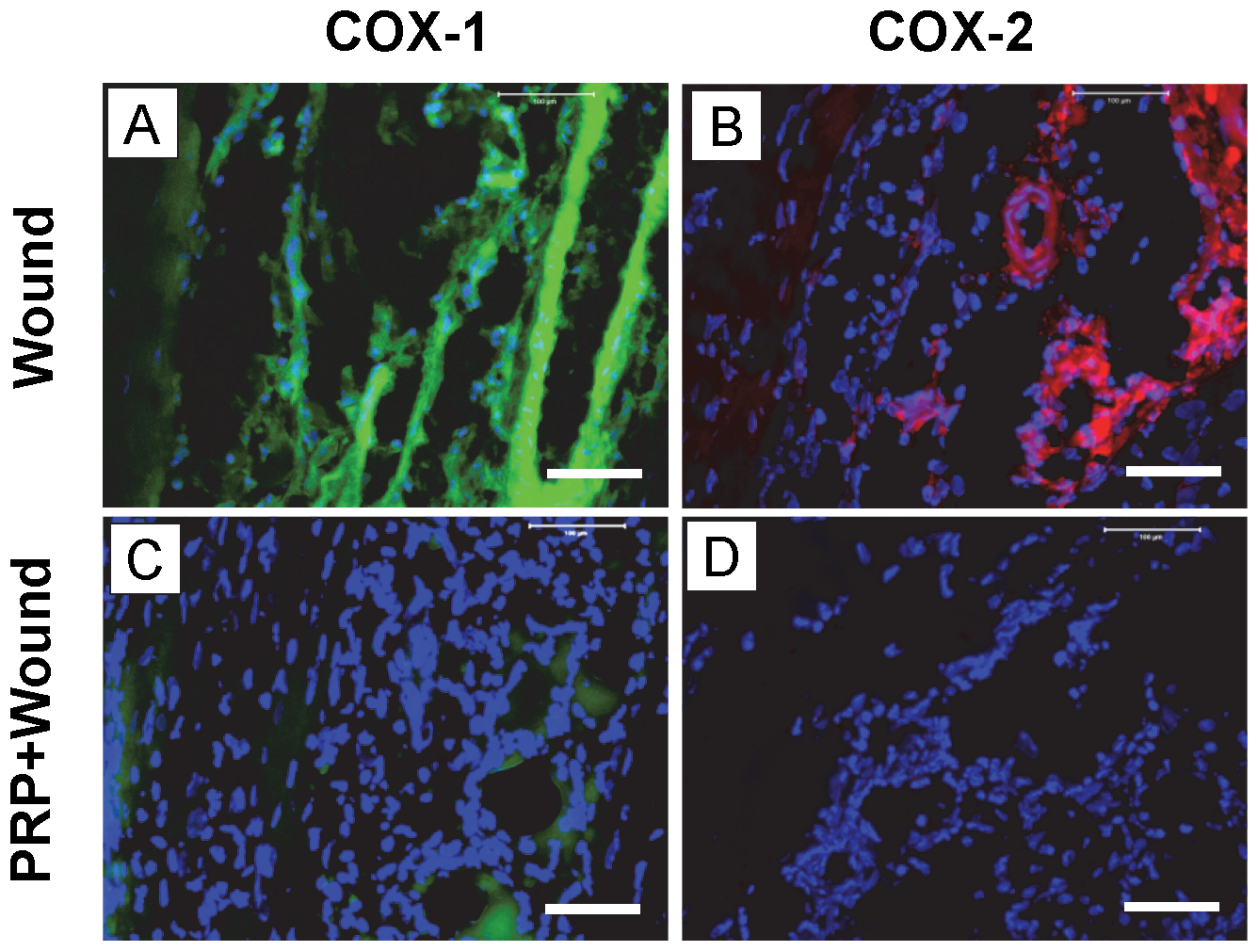
Immunostaining of COX-1 and COX-2 on tissue sections of wounded mouse Achilles on day 3 after wounding and PRP treatment. Dissected tissues were cut, fixed in 4% paraformaldehyde. For COX-1, the tissue sections were stained with goat anti-COX-1 antibody and FITC-conjugated rabbit anti-goat IgG antibody. For COX-2, the tissue sections were stained with goat anti-COX-2 antibody and Cy-3 conjugated donkey anti-goat IgG antibody. Green represents COX-1; red represents COX-2; and blue represents nuclei stained with Hoechst 33342. Both COX-1 (**A**) and COX-2 (**B**) were positively stained on the wound sites of the mouse tendons without PRP treatment. However, treatment with PRP, with its platelet concentration about 4 times over that of mouse whole blood (**Figure** **9**), nearly abolished COX-1 (**C**) and COX-2 (**D**) expressions. Bar: 100 µm.

**Figure 12:**
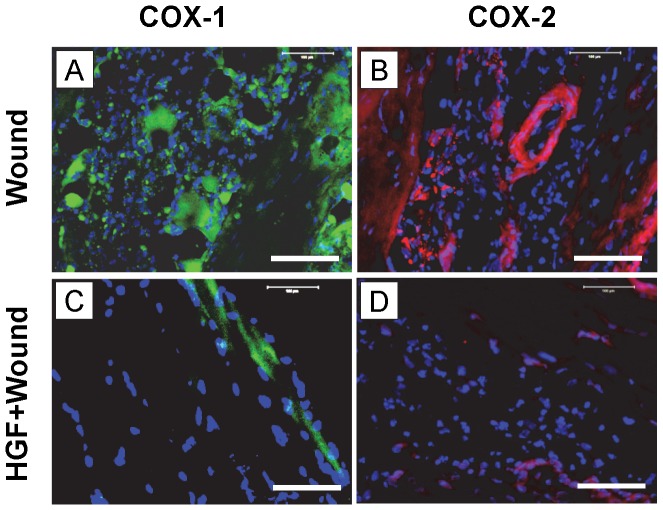
Immunostaining of COX-1 and COX-2 on tissue sections of wounded mouse Achilles tendons on day 3 after wounding and HGF treatment. Mouse Achilles tendons were dissected 3 days after wounding and with or without HGF treatment followed by mincing and fixing tissues in 4% paraformaldehyde. For COX-1 and COX-2 staining, the tissue sections were stained with goat anti-COX-1 and goat anti-COX-2 antibodies respectively, and detected using FITC-conjugated rabbit anti-goat IgG antibody (COX-1) or Cy-3 conjugated donkey anti-goat IgG antibody (COX-2) respectively. Green represents COX-1; red represents COX-2; and blue represents nuclei stained with Hoechst 33342. Both COX-1 (**A**) and COX-2 (**B**) were positively stained on the wounded area without HGF treatment. However, HGF treatment (3 ng in 10 µl saline) decreased COX-1 (**C**) and COX-2 (**D**) expression. Bar: 100 µm.

Stained tissue sections showed that both COX-1 and COX-2 expression were markedly increased in wounded Achilles tendons day 3 after wounding (**Figure 11A, B**). However, 3 days after the injection of 10 µl PRP into wounded tendons, COX-1 and COX-2 expression levels were markedly decreased (**Figure 11C, D**). Further, injection of 3 ng HGF in 10 µl saline into wounded mouse Achilles tendons (**Figure 12A, B**) also decreased COX-1 and COX-2 expression on day 3 post-treatment (**Figure 12C, D**).

## Discussion

Injured tendons are difficult to repair and are susceptible to re-injury, as healing typically results in the formation of mechanically-inferior scar tissue. In fact, the restoration of normal structure and function to injured tendons is one of the most challenging tasks in orthopaedic surgery and sports medicine [30]. Currently, autologous PRP has been adopted as a promising treatment option for tendon injury. However, the efficacy of PRP treatment is highly controversial [8], [31], [32]. We suggest that this confusion partially stems from the fact that the mechanism of PRP’s effects on the inflammatory response of injured tendons is poorly defined. This study was designed to determine the potential effects of PRP treatment on tendon inflammation following injury in an animal model. Using both *in vitro* and *in vivo* models, this study demonstrated that PRP and HGF treatments suppressed the IL-1β-induced gene expression of COX-1, COX-2, and mPGES-1, the protein expression of COX-1 and COX-2, and the production of PGE_2_ in tendon cell culture. Finally, the use of HGF antibody with PRP or HGF could block PRP/HGF’s suppressive effects on tendon cell inflammation. Taken together, the findings of this study indicate that PRP exerts an anti-inflammatory effect on injured tendons, which is mediated, at least in part, by HGF.

As a mediator of PRP’s anti-inflammatory action, as shown in this study, HGF is a pleiotropic factor and plays an essential role in the regulation of cell proliferation, survival, and differentiation in various organs [33]. Our finding that rabbit PRP contained high levels of HGF is consistent with the established facts on PRP. HGF is not only contained within PRP, but is also produced by human tendon cells [34] and human bone marrow stem cells in culture [35]. Therefore, it is likely that HGF was also produced in our tendon cell cultures, and newly produced HGF could add to the blocking effects of exogenously added HGF.

Moreover, HGF is known to function both as an anti-inflammation agent and as a remarkable anti-fibrotic regulator [36]. Its anti-inflammatory action is primarily mediated by the disruption of transcription factor NF-κB signaling, which is a critical regulator of inflammation. This was demonstrated both *in vivo* and *in vitro* in renal inflammation of mouse [33] and human chondrocytes *in vitro* [37]. This mechanism of HGF action was presumably responsible for the anti-inflammatory effects of HGF on COX-1, COX-2, and PGE_2_ production observed in this study. In this regard, the anti-inflammatory effects of PRP and HGF have mechanisms similar to those of NSAIDs, which inhibit COX expression and, as a result, PGE_2_ production in injured tissues. We suggest that the findings of this study may explain how PRP treatment of tendon injuries in human subjects effectively improves pain and function in a clinical setting: PRP likely reduced tendon inflammation and, consequently, alleviated pain, which resulted in improved functional scores in tendon injury patients [17], [18].

It is well established that COX, especially COX-2, is a major marker of tissue inflammation [38], [39]. COX converts arachidonic acid (AA) into prostaglandins, which are further converted into PGE_2_ by PGE_2_ synthase (PGES), including mPGES. PGE_2_ induces vasodilation, vascular permeability, and nociception, and is a major mediator of acute inflammation and pain in tendons and other tissues [2], [40], [41]. Furthermore, high levels of PGE_2_ not only result in exacerbated inflammation, but also suppress matrix synthesis [42]. For example, treatment of human tenocytes with PGE_2_ in culture decreases cell proliferation and collagen production [43], induces aberrant differentiation of TSCs into non-tenocytes *in vitro* [44] and induces degenerative changes in rabbit tendons [45]. Thus, the suppressive effects of PRP on COX expression and PGE_2_ production shown in this study will provide multiple benefits: PRP can not only reduce tendon inflammation and associated pain in clinics, but can also promote the repair of injured tendons. In addition, growth factors contained in PRP can produce stimulatory effects on tendon healing and the fibrin gel in PRP could serve as a natural scaffold to attract cells and, as a result, accelerate tendon healing.

It is known that PRP may contain variable levels of IL-1 receptor antagonist (IL-1ra), as shown in human serum [46]. IL-1ra may block the induction of cellular inflammation in culture by exogenous IL-1β. Additionally, HGF could upregulate IL-1ra expression, thus exerting its anti-inflammatory effects on the tendon cells [20]. However, this potential effect of serum IL-1ra on tendon cell inflammation in our culture experiments must be much smaller than the effects of HGF, because the combined use of PRP and HGF antibody restored more than 60–70% of COX-1 and COX-2 expressions (**Figures 2A** **,** **3A**) and PGE_2_ production (**Figure 5A**), which was induced by IL-1β treatment of tendon cell culture, to levels comparable with PRP treatment alone. Therefore, we suspect that in normal serum derived from healthy animals (rabbits and mice in this study), IL-1ra levels are either low, or may be degraded during the process of PRP preparation, or a combination of the two.

An interesting finding in our *in vivo* model is that while PPP injection did not significantly reduce PGE_2_ levels, it apparently caused a small reduction of PGE_2_ production in wounded mouse Achilles tendons on days 0, 1, 3, and 5 post-treatment (**Figure 10A**). This effect is similar to PRP, although much smaller, probably due to the much lower number of platelets in PPP than in PRP (**Figure 9**). In addition, PPP could possibly contain certain serum factors exerting an inhibitory effect on PGE_2_ production in the wounded tendons. This finding suggests that PRP’s effects on the inflammation of injured tendons are platelet concentration-dependent, and that blood clots, which contain fewer concentrated platelets than PRP, but a higher number of platelets than PPP, can exert anti-inflammatory effects at injured tissue sites. While HGF in mouse PRP was not measured, previous studies have shown that mouse serum contains HGF [47], [48], a finding consistent with our *in vitro* experiments. HGF in mouse PRP and PPP may mediate the anti-inflammatory effects observed in our *in vivo* experiments.

Now a further explanation of the cell culture model used in this study may be necessary to enable a better understanding of our experimental results. First, when tendons are injured, inflammation ensues, which is characterized by an initial upregulation of IL-1β production in injured sites [49]. IL-1β, which is secreted mainly by macrophages, is a potent inflammatory cytokine that upregulates the expression of other inflammatory mediators, including COX and matrix metallopeptidase. Second, tendon cells are known to respond to IL-1β treatment by upregulating COX expression and increasing production of PGE_2_ [6], [26], [50]. Therefore, the use of IL-1β and tendon cells may serve as an appropriate *in vitro* model to simulate tendon inflammation *in vivo*. However, a limitation of the current *in vitro* model is that mechanical loading was not incorporated. Because patients resume daily activities after PRP injection, mechanical loading placed on injured tendons should be an important factor in assessing the efficacy of PRP treatment on injured tendons. In clinics, training regiments are often prescribed, which may have additive effects on tendon inflammation. Based on this, we suggest that appropriate exercise after PRP treatment is likely beneficial, as small mechanical loading on tenocytes suppresses cellular inflammation [26]; however, intensive exercise should be avoided because large mechanical loading may further worsen cellular inflammation by increasing the production of PGE_2_ [44].

In our *in vivo* mouse model, while injections of PRP significantly reduced the production of PGE_2_, they did not completely suppress PGE_2_ levels. This may be attributed to a few factors. The first is the PRP composition. In the PRP preparations, we did not separate white blood cells (WBCs) from platelets because of the small volume of mouse blood. Therefore, our PRP preparations likely contained inflammatory agents (e.g. IL-1β and TNF-α) released from WBCs, which may cause exacerbated inflammation. The second reason may be the PRP concentration used in the animal experiment. Only one dosage of PRP (∼ 4 times the platelet number of mouse whole blood) was used for injection. It is possible that the use of higher doses of PRP and, as a result, higher levels of HGF, would further reduce tendon inflammation marked by high levels of PGE_2_. However, the complete suppression of PGE_2_ production is not desirable, since under normal conditions, tendons contain a basal level of PGE_2_ [44]. This PGE_2_ may play an important role in tendon remodeling [51].

In our *in vivo* study, we used acutely injured tendons of young mice as an experimental model to investigate the anti-inflammatory effects of PRP. The disadvantage with this model is that it does not replicate the chronic tendon inflammation and/or degeneration typical in chronic tendon injury or tendinopathy [52]. Future research should evaluate the effects of PRP treatment on tendinopathy in an animal model. However, it should be noted that the anti-inflammatory effects produced by PRP injection and mediated by HGF, as shown in this study, should be independent of acute or chronic tendon injuries.

It should be stressed that this study was intended to determine whether PRP’s use to treat tendon inflammation had a sound “theoretical basis” (specifically, this study hoped to identify PRP’s specific anti-inflammatory mechanism), which turned out to be the case as revealed both by our *in vitro* and *in vivo* data. However, whether PRP therapy *will* be an effective treatment for any particular tendon injury in a clinical setting can be complicated by a number of patient factors, including age, treatment history, disease history, etc. All these factors, which are not accounted for in the *in vitro* and *in vivo* models of this study, are expected to create huge variations in PRP treatment outcomes [53]. This is particularly true for the general patient population, as opposed to the most active and highly-competitive athletic population. The former is expected to be more variable because of its diverse patient populations, while the later may constitute a more homogenous population.

Clinically, NSAIDs are frequently used in the treatment of tendinopathies to reduce tendon inflammation and associated pain; however, these drugs have serious side effects, ranging from mild symptoms, such as dyspepsia and abdominal discomfort, to more serious adverse events, such as peptic ulcers and life-threatening gastric/duodenal bleeding and perforation [54]. As a result of these side effects, the clinical use of NSAIDs is severely limited. Moreover, while NSAIDs may provide some pain relief to patients, they do not improve the healing process of injured tissues [55] and have been shown to abolish exercise-induced adaptive increases in collagen synthesis in human tendons [56], [57]. On the other hand, PRP is thought to be inherently safe because of its autologous nature. Therefore, the anti-inflammatory function of PRP shown in this study makes PRP an attractive alternative option for treating tendon inflammation and associated pain in clinics.

### Conclusions

This study demonstrates that PRP treatment suppresses tendon cell inflammation *in vitro* and tendon inflammation *in vivo*, marked by the upregulation of COX-1, COX-2, and mPGES-1 expressions, as well as high levels of PGE_2_ production. This anti-inflammatory function of PRP is at least partially mediated through HGF, a major growth factor in PRP, which produces anti-inflammation results similar to PRP. These findings indicate that PRP exerts anti-inflammatory effects on injured tendons, which may explain why PRP treatments in clinics can improve tendon function in patients with tendon injury. Future research is required to determine optimal PRP dosage regimens, to evaluate the different effects produced by varied PRP compositions, and to evaluate the interactive effects between PRP treatment and exercise regimens (or mechanical loading) on injured tendons.
